# Effect of Cysteine Mutations in the Extracellular Domain of CM2 on the Influenza C Virus Replication

**DOI:** 10.1371/journal.pone.0060510

**Published:** 2013-04-04

**Authors:** Yasushi Muraki, Takako Okuwa, Toshiki Himeda, Seiji Hongo, Yoshiro Ohara

**Affiliations:** 1 Department of Microbiology, Kanazawa Medical University School of Medicine Uchinada, Ishikawa, Japan; 2 Department of Infectious Diseases, Yamagata University Faculty of Medicine Iida-Nishi, Yamagata, Japan; University of Edinburgh, United Kingdom

## Abstract

CM2 is the second membrane protein of influenza C virus and possesses three conserved cysteines at residue 1, 6 and 20 in its extracellular domain, all of which are involved in the formation of disulfide-linked oligomers of the molecule. In the present study, to examine the effect of CM2 oligomerization on virus replication, we generated a mutant recombinant virus, rC1620A, in which all three cysteines on CM2 were substituted to alanines. The rC1620A virus was more attenuated than the recombinant wild-type (rWT) virus in cultured cells. The CM2 protein synthesized in rC1620A-infected cells could not apparently be detected as a tetramer and was transported to the cell surface less efficiently than was authentic CM2. The amount of CM2 protein incorporated into the rC1620A virions was comparable to that into the rWT virions, although the main CM2 species in the rC1620A virions was in the form of a dimer. Analyses of one-step grown virions and virus-infected cells could not provide evidence for any difference in growth between rC1620A and rWT. On the other hand, the amount of genome present in VLPs possessing the mutant CM2 (C1620A-VLPs) was approximately 31% of that in VLPs possessing wild-type CM2 (WT-VLPs). The incoming genome from VLPs was less efficiently transported to the nucleus in the C1620A-VLP-infected cells than in WT-VLP-infected cells, leading to reduced reporter gene expression in the C1620A-VLP-infected cells. Taken together, these findings demonstrate that CM2 oligomerization affects the packaging and uncoating processes. Thus, we concluded that disulfide-linked CM2 oligomers facilitate virus growth by affecting the replication processes.

## Introduction

RNA segment 6 (M gene) of influenza C/Ann Arbor/1/50 is 1,180 nucleotides in length and encodes the M1 and CM2 proteins [1], [2]. The predominant mRNA lacks a region from nucleotides 754 to 981, and encodes a 242-amino-acid matrix protein, M1 [3]. Unspliced mRNA from the RNA segment 6 (a collinear transcript of the gene) that is synthesized in small quantities encodes the P42 protein, which contains an additional 132 amino acids on the C-terminus of M1 [4], [5]. P42 is cleaved by a signal peptidase at an internal cleavage site to generate CM2 composed of the C-terminal 115 amino acids, in addition to the M1’ protein composed of the N-terminal 259 amino acids [6], [7].

The biochemical characteristics of CM2 have been precisely analyzed. CM2 is a type III membrane protein that is oriented in membranes with a 23-amino-acid N-terminal extracellular domain, a 23-amino-acid transmembrane domain, and a 69-amino-acid C-terminal cytoplasmic domain [8], [9]. It is abundantly expressed in virus-infected cells and a small amount of CM2 is incorporated into the virus particles [8]. It forms disulfide-linked dimers and tetramers, and is post-translationally modified by N-glycosylation, palmitoylation and phosphorylation [8]–[10].

CM2 forms a Cl^–^ channel when expressed in *Xenopus laevis* oocytes [11]. Electrophysiological studies of CM2-expressing mouse erythroleukemia cells have identified proton and Cl^–^ permeabilities (Muraki Y, Chizhmakov IV, Ogden DC, Hay A, unpublished data). When expressed together with a pH-sensitive hemagglutinin of influenza A virus, CM2 was demonstrated to modulate the pH of the exocytic pathway, suggesting that CM2 has proton permeability [12].

To clarify the role(s) of CM2 in virus replication, virus-like particles (VLPs) and recombinant influenza viruses possessing CM2 mutants have been analyzed. The packaging and uncoating processes of the CM2-deficient influenza C VLPs were found to be impaired [13]. A recombinant influenza C virus lacking CM2 palmitoylation had no defects in growth properties [14], whereas the growth of a CM2 glycosylation-deficient influenza C virus was impaired [15]. A chimeric influenza A virus M2 protein containing the CM2 transmembrane domain, not authentic CM2, could partially restore the infectious virus production of an M2-deficient influenza A virus [16]. Taken together, the role(s) of CM2 in virus replication remains to be fully elucidated, particularly in terms of the contribution of proton and Cl^–^ permeabilities to the virus replication.

The cysteines at residue 1, 6 and 20 in the extracellular domain of CM2 are evolutionarily conserved among the influenza C virus isolates examined to date [17], [18]. Analyses of COS cells expressing CM2 mutants in which the three cysteines were individually or in combination substituted to alanines showed that all of the cysteines can participate in the formation of disulfide-linked dimers and/or tetramers, and that disulfide bond formation, although not essential for proper oligomerization, may stabilize the CM2 multimer [19]. However, the significance of the cysteines in virus replication remains to be clarified.

In the present study, to elucidate the role(s) of CM2 oligomerization in the influenza C virus replication cycle, we generated a recombinant influenza C virus and VLPs in which the three cysteines in the extracellular domain of CM2 were substituted to alanines. As a result, we found that CM2 oligomerization by disulfide-bonding was not essential but required for efficient virus replication.

## Materials and Methods

### Ethics Statement

Regarding the use of HMV-II cells, the authors consulted the Kanazawa Medical University Research Ethics Committee and have received a formal written waiver from the Committee. HMV-II and LLC-MK_2_ cells were provided by the Department of Infectious Diseases, Yamagata University Faculty of Medicine, Japan. The preparation of LLC-MK_2_ cells was published previously by our research group [14], [15]. 293T cells were provided by Dr. Yoshihiro Kawaoka (Institute of Medical Science, University of Tokyo, Japan). We did not conduct research outside Japan. As corresponding author, I have obtained written informed consent from the coauthors. The Kanazawa Medical University Ethics Committee approved this consent procedure.

### Cells, Antibodies and Viruses

HMV-II cells were maintained in RPMI 1640 medium with 10% calf serum [20]. LLC-MK_2_ cells were maintained in minimal essential medium with 5% fetal bovine serum and 5% calf serum [14]. 293T cells were maintained in Dulbecco’s modified Eagle’s medium with 10% fetal bovine serum [21]. Monoclonal antibodies (MAbs) against the HEF (J14, D37, S16), NP (H27, H31) and M1 (L2) proteins of C/Ann Arbor/1/50 (AA/50), and antisera against the CM2 protein were prepared as described previously [4], [22], [23]. Anti-α-actin and anti-EGFP polyclonal antibodies were purchased from Sigma (St. Louis, MO) and Thermo Fisher Scientific Inc. (Waltham, Mass.), respectively. A stock of AA/50 was propagated in 9-day old chicken eggs as described previously [24].

### Plasmid DNAs

The Pol I plasmids for the expression of virus RNAs (vRNAs) of AA/50 (pPolI/PB2, pPolI/PB1, pPolI/P3, pPolI/HEF, pPolI/NP, pPolI/M, and pPolI/NS), and the plasmids for the expression of the influenza C virus proteins (pcDNA/PB2-AA, pcDNA/PB1-AA, pcDNA/P3-AA, pME18S/HEF-AA, pCAGGS.MCS/NP-AA, pCAGGS.MCS/M1-AA, pME18S/Met-CM2-YA, pME18S/NS1-YA, and pME18S/NS2-YA) were reported previously [21], [25]. The pPolI/CM2-C1620A plasmid, used to generate virus RNA (vRNA) encoding M1 and CM2-C1620A (see below), was generated based on pPolI/M. The pPolI/NP-AA.GFP(−) and pPolI/NP-AA.Luc(−) plasmids were described previously [21]. The pME18S/CM2-C1620A plasmid for the expression of the mutant CM2 protein (CM2-C1620A), in which the cysteines at residue 1, 6 and 20 were substituted to alanines, was constructed based on pPolI/CM2-C1620A. Details of the primers and PCR protocols used will be provided on request.

### Reverse Genetics of Influenza C Virus

Reverse genetics of influenza C virus was performed as described previously [25]. Briefly, to rescue the recombinant wild-type (rWT) virus, the seven Pol I plasmids and nine expression plasmids described above were transfected into 293T cells. The culture medium harvested at 72 h posttransfection (p.t.) was then inoculated into 9-day old chicken eggs. For the generation of a CM2 mutant virus (rC1620A), pPolI/CM2-C1620A, instead of pPolI/M, was transfected into 293T cells together with the 15 remaining plasmids.

### Determination of the Infectious Titers of Viruses

The infectious titers of a stock of the recombinant viruses and the supernatants of recombinant-infected HMV-II or LLC-MK_2_ cells were determined according to the procedure reported previously [14], [26]. Briefly, monolayered LLC-MK_2_ cells infected with the viruses were reacted with anti-HEF MAb D37 (primary antibody) and anti-mouse IgG conjugated with HRP (secondary antibody) (BioRad, Hercules, CA), and the virus plaques were visualized using True Blue® (KPL, Gaithersburg, MD) substrate.

### Radioimmunoprecipitation

HMV-II cells infected with recombinant viruses were labeled with [^35^S]methionine (ARC) (30 µCi/35 mm dish) for 20 min at 26 h postinfection (p.i.) in RPMI 1640 medium lacking methionine. In the pulse-chase experiments, the infected cells labeled with [^35^S]methionine were chased for the indicated periods. Cells were then disrupted and subjected to immunoprecipitation with MAbs against HEF (J14), NP (H27) and M1 (L2) or anti-CM2 serum as described previously [4]. The immunoprecipitates obtained were then analyzed by SDS-PAGE on 17.5% gels containing 4 M urea, and processed for fluorography [24].

Chemical cross-linking was performed as described previously [19]. Briefly, the infected cells were labeled for 20 min at 26 h p.i. and chased for 2 h. A stock solution of 100 mM dithiobis (succinimidylpropionate) (DSP) prepared in DMSO was diluted in PBS to 0.5, 2.5, and 12.5 mM and then added to the infected-monolayers. After incubation overnight at 4°C, 50 mM glycine was added to neutralize excess cross-linker. The cells were then lysed in the presence of 50 mM iodoacetamide and immunoprecipitated with anti-CM2 serum, followed by SDS-PAGE under non-reducing conditions.

### Cell Surface Biotinylation

Detection of virus proteins expressed on the infected cells was performed as described previously [15], [27]. Briefly, HMV-II cells infected with the recombinants were washed with ice-cold PBS at 26 h p.i. and exposed to 0.5 mg/ml of sulfo-NHS-LC-LC-biotin (Thermo Fisher Scientific Inc.) for 30 min on ice. The reaction was stopped by rinsing the cells twice with 100 mM glycine in ice-cold PBS, and the cells were then lysed in 450 µl of RIPA buffer containing a cocktail of protease inhibitors and incubated for 30 min on ice. The resulting lysates were centrifuged at 12,000 rpm for 20 min at 4°C. The supernatants (420 µl) were incubated for 60 min at room temperature with streptavidin-agarose (Thermo Fisher Scientific Inc.) to isolate biotinylated proteins by precipitation with streptavidin-agarose. The precipitates obtained (biotinylated proteins) and whole cell lysates (30 µl) were respectively subjected to immunoblotting.

### RNA Extraction, Reverse Transcription, and Real-time PCR

The RNAs were extracted from the VLP-infected HMV-II cells, recombinant viruses or VLPs using an RNeasy Mini Kit (Qiagen, Hilden, Germany). The RNA preparation was treated with DNase I (Takara) and then reverse transcribed using a primer complementary to nucleotide positions 1 to 12 of vRNA [13], [28] or the GFP gene [15]. The cDNA was then subjected to real-time PCR using a pair of primers specific to the influenza C virus NS gene (5′-GCTTCTATTCAACGGGACGA-3′ and 5′-TTGGTGCTATGTTTCTTGGA-3′) or GFP gene [13], [15]. The real-time PCR was carried out on an ABI Prism 7900 HT Fast Real-Time PCR System (Applied Biosystems, Carlsbad, CA) using a Power SYBR Green PCR Master Mix Kit (Applied Biosystems) according to the manufacturer’s instructions. The standard curve was calculated based on the results of real-time PCR using a series of 10-fold dilutions of pPolI/NS or pPolI/NP-AA.GFP(−) as a template. As a loading control for RNAs extracted from the cells, β-actin mRNA was quantified by real-time PCR as described previously [13]. The details of primers and the PCR protocols used will be provided on request.

### Immunoblotting

Immunoblotting for the plasmid-transfected 293T cells, and purified virions and VLPs were carried out as described previously [13], [21] using the MAbs (S16, H31, L2) and anti-CM2 serum described above. Band intensities were measured using ImageJ 1.42 q software.

### Generation, Purification and Infection of VLPs

WT-VLPs were generated and purified as described previously [13], [21]. For the generation of C1620A-VLPs, 293T cells were transfected with the same combination of plasmids as for the WT-VLPs, except that pME18S/CM2-C1620A was used instead of pME18S/Met-CM2-YA. Protein concentrations of the purified VLPs were determined using Pierce 660 nm Protein Assay Reagent (Thermo Fisher Scientific Inc.).

The purified VLPs were treated with N-tosyl-L-phenylalanyl chloromethyl ketone (TPCK)-treated trypsin (20 µg/ml) at 37°C for 10 min, followed by the addition of soybean trypsin inhibitor. The monolayered HMV-II cells were infected with the VLPs at 33°C for 60 min and subsequently infected with the helper virus (AA/50) at a multiplicity of infection (MOI) of 5, and incubated for the indicated periods. For the quantification of incoming GFP-vRNA (see below), the helper virus was not used for infection to avoid the replication of GFP-vRNA in the infected cells. Luciferase (Luc) activity in the VLP-infected cells was measured using a GloMax 96 Microplate Luminometer (Promega, Madison, WI).

### Flow Cytometry of Recombinant- and VLP-infected HMV-II Cells

Flow cytometry of recombinant-infected HMV-II cells was performed as described previously [13], [15]. Briefly, monolayered HMV-II cells were trypsinized and then added to the recombinant viruses. After incubation on ice for 30 min, an aliquot of the cells was transferred to 33°C and incubated for 180 min. The cells were washed and incubated with the anti-HEF MAb J14 (primary antibody) and FITC-conjugated anti-mouse IgG antibody (secondary antibody) (Jackson ImmunoResearch Europe, Ltd., UK). The cells were subjected to flow cytometry using a FACSCalibur (Becton Dickinson, San Jose, CA).

### Cell Fractionation

VLP-infected HMV-II cells were subjected to fractionation as described previously [13], [15], [29]. Briefly, HMV-II cells mildly solubilized with RSB buffer (10 mM Tris-HCl, 100 mM NaCl, 1.5 mM MgCl2, pH 8.0) containing 0.3% NP-40 for 30 min at 0°C were divided into two fractions by centrifugation at 1,200×g for 5 min at 4°C. The precipitate was washed twice with the RSB buffer containing 0.3% NP-40 and then used as the nuclear fraction. The supernatant was re-centrifuged at 10,000×g for 5 min at 4°C, and the resulting supernatant was used as the cytoplasmic fraction.

### Statistical Analysis

Data between groups were analyzed using a paired *t*-test. A p value of less than 0.05 was considered statistically significant.

## Results

### Generation of a Recombinant Influenza C Virus Possessing CM2 Mutations

There are three conserved cysteines at residues 1, 6 and 20 in the extracellular domain of CM2 [17], [18]. Li *et al.* precisely analyzed the roles of the cysteines in the CM2 oligomerization, transportation and cell surface expression using plasmid-transfected COS cells expressing a series of CM2 cysteine mutants [19]. As a result, all of the cysteines were shown to be involved in multimer formation of the CM2 molecules. In particular, a mutant protein, CM2-C1620A, in which all three cysteines were substituted to alanines, lacked the ability to form disulfide-linked oligomers, although it was transported to the cell surface. Based on these findings, in the present study we therefore generated a recombinant influenza C virus (rC1620A) in which all of the cysteines of CM2 were substituted to alanines to investigate the effect(s) of CM2 oligomerization on virus replication.

As described in the Materials and Methods section, the pPolI/CM2-C1620A plasmid was transfected to 293T cells together with the six remaining Pol I plasmids and nine protein-expressing plasmids. The supernatant of the transfected cells was inoculated into embryonated chicken eggs, and a stock of the recombinant (rC1620A) was obtained.

### Growth of the Recombinant C1620A Virus

**Figure 1:**
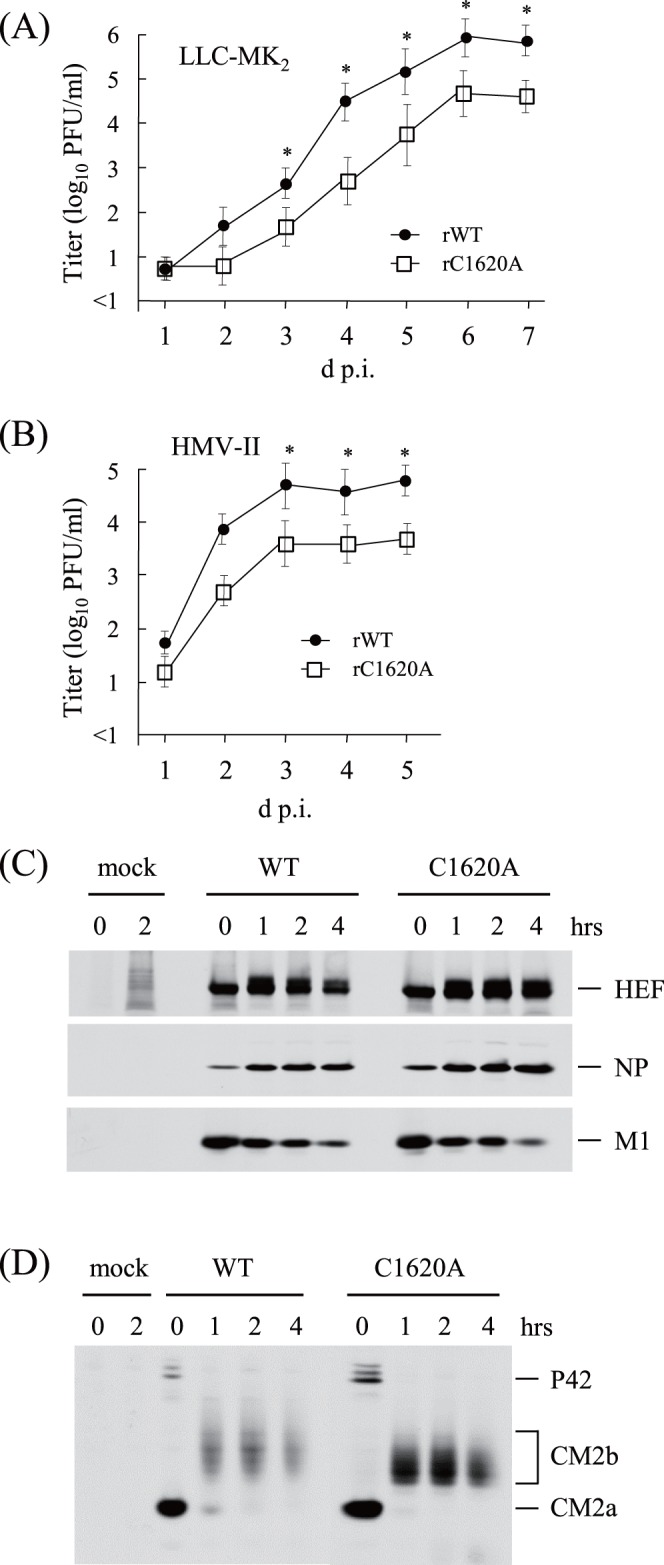
Growth kinetics and protein synthesis of the recombinant viruses. (A) (B) LLC-MK_2_ (A) or HMV-II (B) cells were infected with the recombinants at an MOI of 0.001 and incubated at 33°C in the presence of trypsin (10 µg/ml) for the indicated periods (days). The virus yield in the culture media was titrated on LLC-MK_2_ cells. Data are expressed as the mean ± standard deviation (SD) in three independent experiments. All comparisons between groups were statistically evaluated (*p<0.05). (C) (D) Mock- or virus-infected HMV-II cells were pulse-labeled with [^35^S]methionine at 26 h p.i. and chased for the indicated periods (hrs). The cells were lysed, immunoprecipitated with MAbs against HEF, NP and M1 (C) or anti-CM2 serum (D), and analyzed by SDS-PAGE under reducing conditions. CM2 is modified by N-glycosylation on an asparagine at residue 11 [9]. CM2a possesses a mannose-rich oligosaccharide core on the residue. The maturation of the carbohydrate chain from the high-mannose type to the complex-type converts CM2a into CM2b, with the latter modified by addition of polylactosaminoglycan [8], [9].

To investigate the effect(s) of cysteine mutations on virus replication, we infected LLC-MK_2_ or HMV-II cells with the rC1620A virus at an MOI of 0.001, and the cells were incubated at 33°C in the presence of trypsin for 7 or 5 days, respectively. Differences in growth between rWT and rC1620A were observed in both LLC-MK_2_ and HMV-II cells (Fig. 1A, B). The virus yields of rWT were significantly higher than those of rC1620A after 3 days p.i. (p<0.05). Furthermore, we confirmed that there were no unwanted mutations in the M gene of rC1620A harvested at 7 (LLC-MK_2_) and 5 (HMV-II) days p.i. (data not shown). These findings suggest that the CM2 cysteine mutations introduced into the recombinants affect virus replication.

### Synthesis of Virus Proteins in the Infected Cells

The infected cells were labeled with [^35^S]methionine, chased, and then immunoprecipitated with MAbs against HEF, NP and M1, and anti-CM2 serum, followed by SDS-PAGE under reducing conditions. No apparent differences were observed in the synthesis and maturation of HEF, NP and M1 (Fig. 1C), nor in the synthesis and maturation of CM2 (Fig. 1D); CM2a, possessing a mannose-rich oligosaccharide core, in the pulse sample (lane 0) properly matured to CM2b possessing a complex-type carbohydrate chain in both cell populations. Measurement of the band intensities of the pulse-labeled CM2a (lane 0) and the CM2b proteins in the chased samples (lanes 1, 2 and 4) revealed no apparent differences in the ratios of CM2a to CM2b between the rWT- and rC1620A-infected cells (data not shown). Thus, it is unlikely that the introduced mutations affect the stability of CM2 synthesized in the virus-infected cells.

We also examined the effect of the CM2 mutations on the cleavage efficiency of P42 and the splicing ratio of the M gene. The cleavage of P42 can be calculated as the ratio of CM2a to P42 just after pulse labeling, as reported previously [15]. The ratio of CM2a to P42 in the rWT-infected cells (1.0∶0.1) was virtually identical to that in the rC1620A-infected cells (Fig. 1C and 1D), suggesting that the introduced mutation did not affect P42 cleavage. The splicing ratio of the influenza C virus M gene is correlated with the ratio of M1 to CM2a just after pulse labeling, as reported previously [30]. Fig. 1(C) and 1(D) show that there was no significant difference in the ratio of M1 to CM2a between rWT- and rC1620A-infected cells (1.0∶0.7), suggesting that the mutations introduced into the M gene did not affect the balance of spliced/non-spliced M gene mRNA.

**Figure 2:**
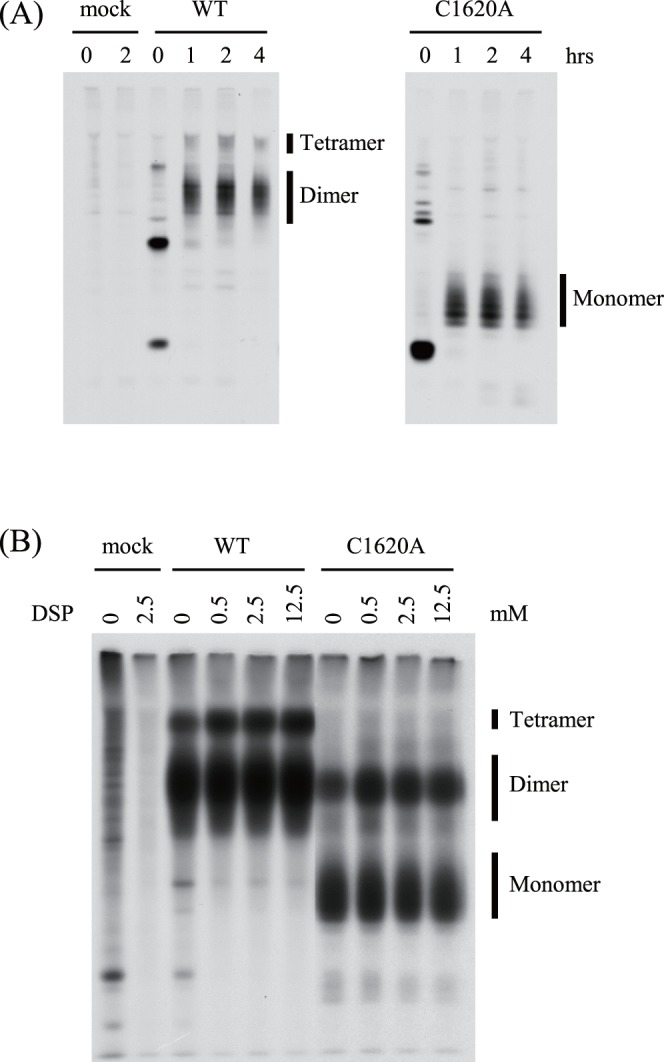
Oligomerization of CM2 in virus-infected cells. (A) Mock- or virus-infected HMV-II cells were pulse-labeled with [^35^S]methionine at 26 h p.i. and chased for the indicated periods (hrs). The cells were lysed, immunoprecipitated with anti-CM2 serum, and analyzed by SDS-PAGE under non-reducing conditions. (B) Mock- or virus-infected cells were pulse-labeled at 26 h p.i. with [^35^S]methionine and chased for 2 h. The monolayers were then treated with 0, 0.5, 2.5, or 12.5 mM DSP overnight at 4°C, immunoprecipitated with anti-CM2 serum and analyzed by SDS-PAGE under non-reducing conditions.

The CM2 protein synthesized in the virus-infected cells were analyzed under non-reducing conditions (Fig. 2A). The infected cells were lysed in the presence of 50 mM iodoacetamide as described previously [4], [19] and immunoprecipitated with anti-CM2 serum, followed by SDS-PAGE. In the chased samples, the bands corresponding to dimers and tetramers were detected in rWT-infected cells, whereas no dimeric and tetrameric forms of CM2 could be detected in the rC1620A-infected cells. This finding was consistent with that reported for the CM2-C1620A protein expressed in COS cells [19].

In order to examine the possibility that the mutant CM2-C1620A protein may form a dimer or tetramer that is held together weakly by non-covalent forces, an experiment using a chemical cross-linker was performed (Fig. 2B). The virus-infected cells were pulse-labeled with [^35^S]methionine for 20 min at 48 h p.i. and then chased for 2 h. Monolayers of the labeled cells were incubated with DSP (a homobifunctional cross-linking reagent), immunoprecipitated and analyzed by SDS-PAGE under non-reducing conditions. In the WT-infected cells, both dimers and tetramers were clearly detected. In contrast, in the rC1620A-infected cells the cross-linked dimers of CM2-C1620A were detected even in the absence of DSP (approximately 25% that of rWT). It should be noted that the tetrameric form of CM2-C1620A could be detected at less than trace amounts in the presence of DSP. Furthermore, the tetrameric CM2-C1620A could not be detected on film even at longer exposure times (data not shown). Thus, it is unlikely that the CM2-C1620A protein forms a tetramer at a level comparable to that of the authentic CM2 in virus-infected cells.

### Cell Surface Expression of CM2

**Figure 3:**
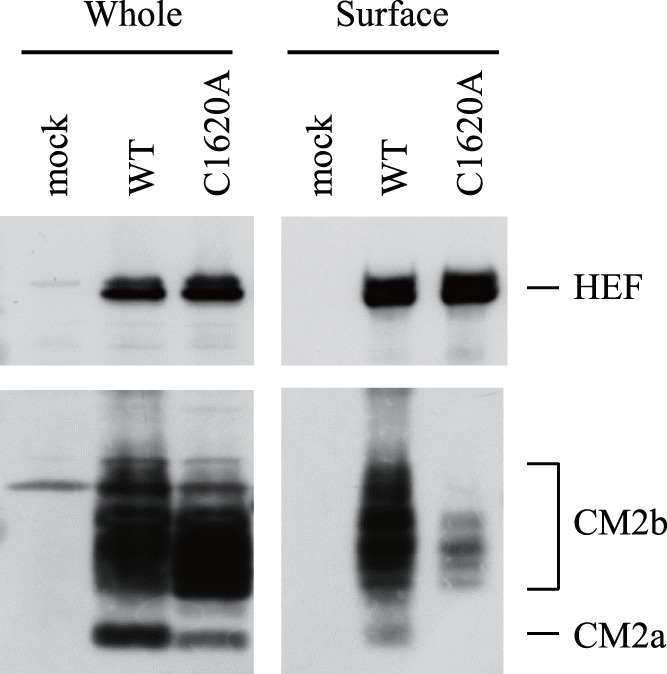
>Cell surface expression of HEF and CM2. The surface of the mock- or virus-infected cells was biotinylated, and the cells were then lysed. The biotinylated proteins were precipitated with streptavidin-agarose. The precipitates (Surface) or whole cell lysates (Whole) were subjected to SDS-PAGE, followed by immunoblotting using anti-HEF MAb (S16) or anti-CM2 serum. CM2a and CM2b indicate the glycosylated forms of CM2 as described in the legend of Fig. 1(D).

As shown in Fig. 1(D) and Fig. 2, the mutant CM2-C1620A protein matured properly in virus-infected cells, but its tetramer formation was impaired. To examine the surface expression of CM2, cell surface proteins of the virus-infected cells were biotinylated, precipitated with streptavidin-agarose and then analyzed by immunoblotting (Fig. 3), as we could not detect CM2 on the surface of the HMV-II cells infected with recombinants by immunofluorescence (Fig. S1). No differences in the amount of surface HEF were observed (Fig. 3, upper panel), whereas the amount of surface CM2-C1620A on the rC1620A-infected cells was less than that of CM2 on rWT-infected cells (Fig. 3, lower panel). Together with the observations shown in Fig. 1(D) and Fig. 2, the result shown in Fig. 3 suggests that the cysteine mutations introduced affect the transport efficiency of CM2; CM2 tetramer formation is required for its efficient transportation.

Immunofluorescence analysis of CM2-expressing COS cells has shown that C1620A-CM2 was transported to the cell surface in a manner similar to that of wild-type CM2 [19]. This discrepancy may be derived from differences in the methods adopted in the study or the higher expression level of CM2 in plasmid-transfected cells than in virus-infected cells (data not shown).

### Effect of CM2 Mutation on Genome Packaging and Uncoating

Furukawa *et al.* have previously shown that CM2 is involved in genome packaging and uncoating using CM2-deficient VLPs (13). In the present study, as we wished to obtain evidence that CM2 is involved in the packaging process using recombinant viruses, not VLPs, one-step grown virions in cultured cells were examined. HMV-II cells were infected with the rWT or rC1620A virus at an MOI of 1 in the absence of trypsin, and progeny viruses generated from the cells were examined. There were no significant differences in growth kinetics between rWT and rC1620A from 1 to 5 days p.i. (Fig. S2). The progeny virions collected at 48 h p.i. were purified by ultracentrifugation through a 30% sucrose cushion, as described previously [13], [15], and the protein concentration was measured. We could not detect any difference in the amount of protein of the purified virions generated from a given number of infected cells (relative ratio; rWT:rC1620A = 1∶1.19).

**Figure 4:**
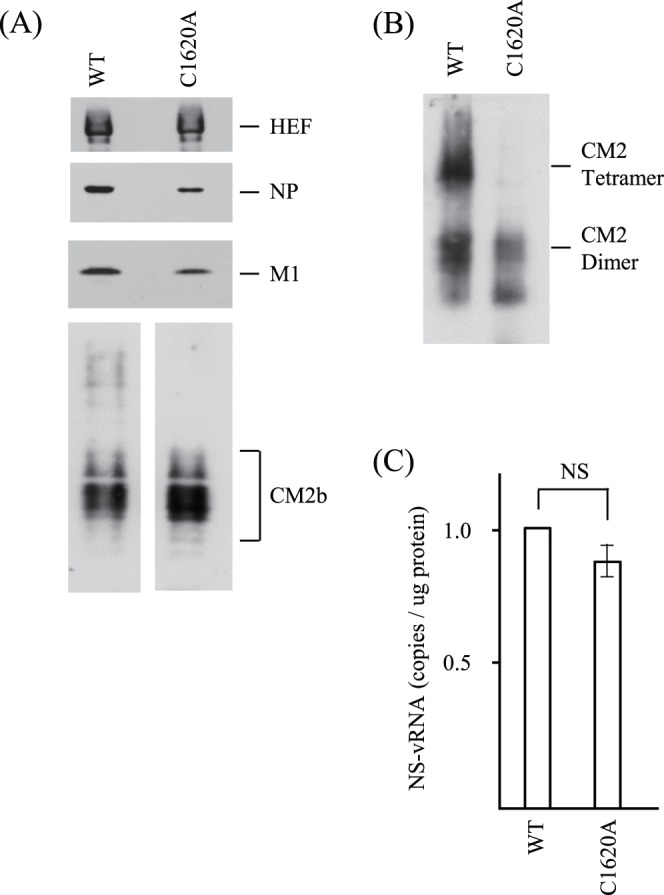
Proteins and genome of recombinant viruses. HMV-II cells were infected with the recombinant viruses at an MOI of 1 and the cells were incubated at 33°C for 48 h in the absence of trypsin. The progeny viruses were purified, and a given amount of the purified virions was subjected to SDS-PAGE under reducing (A) or non-reducing (B) conditions, followed by immunoblotting using MAbs against HEF, NP and M1 and anti-CM2 serum. CM2b indicates the glycosylated form of CM2 as described in the legend of Fig. 1(D). Aliquots of the purified virions were subjected to real-time PCR for the quantification of NS-vRNA (C). The copy number of the NS-vRNA in the WT virus was used for normalization. Each bar represents the mean ± standard errors of the means. NS; not significant.

An equal amount of protein (5 µg) from the respective purified virions was subjected to immunoblotting (Fig. 4A, B). The ratio of NP to M1 was 0.77 for rWT and 0.65 for rC1620A virions (Fig. 4A, upper three panels), suggesting less efficient genome packaging into the rC1620A viruses than into the rWT viruses, as described previously for influenza C VLPs [13], [15]. To examine this possibility, aliquots of the virion preparations were subjected to real-time PCR to quantify the NS gene in the progeny virions (Fig. 4C). There was no significant difference in the amount (copies per µg of virion protein) of the NS gene between the two virion populations, although the amount in rC1620A was reproducibly lower than that in rWT (data not shown). Thus, analyses of the one-step grown viruses could not provide clear evidence for the involvement of CM2 mutation in genome packaging, suggesting that a subtle difference in the high-MOI-infection experiment resulted in a considerable difference in the multi-step growth experiment (Figs. 1A, B).

To investigate whether C1620A-CM2 is incorporated into progeny virions, aliquots of the purified virions were subjected to immunoblotting with anti-CM2 serum under reducing (Fig. 4A) and non-reducing conditions (Fig. 4B). Interestingly, there was no apparent difference in the amount of incorporated CM2 between the rWT and rC1620A viruses (Fig. 4A, lower panel). Under non-reducing conditions, both tetrameric and dimeric forms of CM2 were detected in the rWT virions, whereas the dimeric form was mainly detected in the rC1620A virions (Fig. 4B). The molecular species that migrated faster than the dimers are of unknown origin.

Attempts have been made to analyze the effect of CM2 mutations on the uncoating process of the recombinant viruses. First, to investigate the attachment and internalization of viruses, recombinant-infected cells were subjected to flow cytometry. The HMV-II cells (10^6^ cells) were added to amniotic fluids containing recombinant viruses (6.4 hemagglutinin units), and then analyzed as described previously [15]. As a result, there was no difference in the histograms from the rWT- and rC1620A-infected cells (data not shown), indicating that the attachment and internalization of the rC1620A virus occurred as efficiently as those of the rWT virus.

Next, the amounts of NS-vRNA in the virus-infected cells were compared. Brabec-Zaruba *et al.* reported that the amount of incoming virus RNA decreased (roughly 30% of the input) at 1 to 2 h p.i. in human rhinovirus 2-infected cells [31]. Furukawa *et al.* showed that the amount of incoming GFP-vRNA at 1 h p.i. was lower in the CM2-deficient-VLP-infected cells than in the WT-VLP-infected cells due to the inefficient uncoating of the CM2-deficient-VLPs [13]. Based on these observations, we hypothesized that the amount of NS-vRNA would decrease more dramatically in rC1620A-infected cells than in rWT-infected cells at the early phase of infection if the uncoating of the rC1620A virus was impaired. To this end, HMV-II cells were infected with a stock of the recombinant viruses at an MOI of 0.1, and the infected cells were harvested every 1 h up to 12 h p.i. The RNAs extracted from the respective cells were then subjected to real-time PCR for the quantification of NS-vRNA. No significant differences, however, were observed in the decrease in NS-vRNA between the rC1620A- and rWT-infected cells (data not shown), which directed us to quantify the GFP-vRNA in the VLP-infected cells (see below). Thus, we could not demonstrate the involvement of CM2 in the packaging or uncoating process using recombinant viruses.

### Analysis of VLPs and VLP-infected Cells

As the above-mentioned experiments involving recombinant viruses did not provide us with evidence of differences in growth in cultured cells (Fig. 1A and B), we next analyzed influenza C VLPs. Briefly, to generate wild-type (WT-) VLPs containing GFP-vRNA as the genome, pME18S/Met-CM2-YA (an expression plasmid for wild-type CM2) was transfected into 293T cells together with pPolI/NP-AA.GFP(−) and the other protein-expressing plasmids (for PB2, PB1, P3, HEF, NP, M1, NS1, and NS2). At 48 h p.t., the generated WT-VLPs in the culture media were collected and purified as described previously [13], [15]. To obtain VLPs possessing the mutant CM2 protein (C1620A-VLP), pME18S/CM2-C1620A, instead of pME18S/Met-CM2-YA, was transfected together with the nine other plasmids. As a control, CM2-deficient VLPs (ΔCM2-VLP) were generated by omitting pME18S/Met-CM2-YA from the plasmid mixture for WT-VLPs. The amount of protein of the purified WT-VLPs generated from a given number of VLP-producing 293T cells was identical to that of C1620A-VLPs (WT-VLP:C1620A-VLP = 1.00∶1.05), which is consistent with our previous finding that CM2 mutation did not affect the efficiency of VLP formation [13], [15].

**Figure 5:**
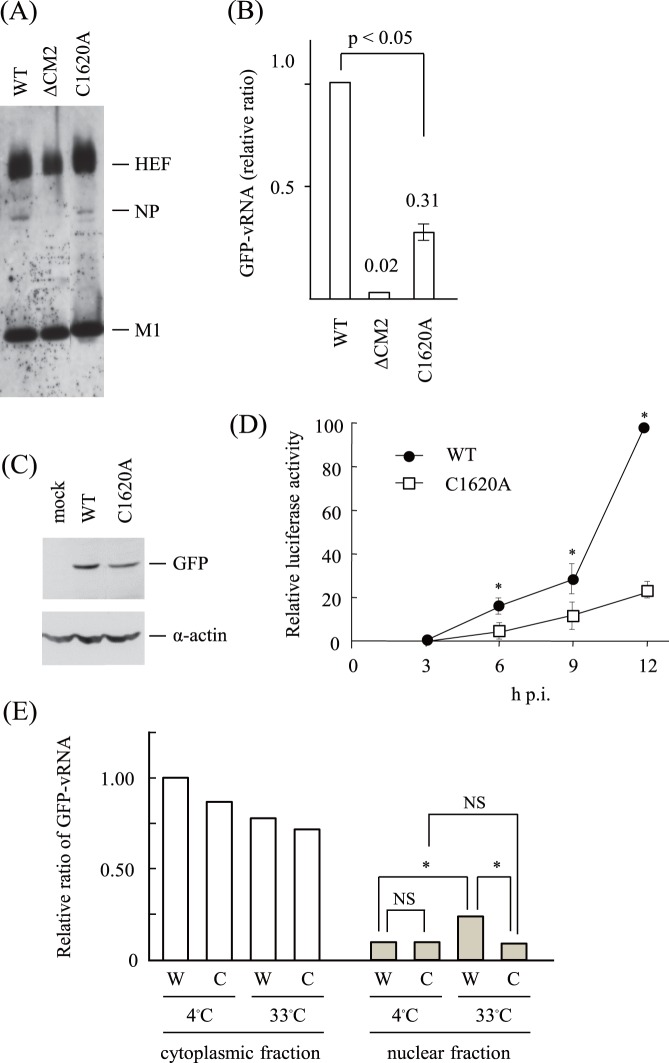
Proteins and gene expressions in VLPs and VLP-infected cells. (A) (B) WT-, CM2-deficient (ΔCM2)- or C1620A-VLPs were generated and purified as described in Materials and Methods. The VLPs were subjected to immunoblotting using a mixture of MAbs against HEF, NP and M1 (A), or to real-time PCR for the quantification of GFP-vRNA (B). The copy number of the GFP-vRNA in the WT-VLPs was used for normalization. The data obtained from three independent experiments were statistically evaluated using a paired *t*-test. (C) (D) HMV-II cells infected with mock, WT-VLPs or C1620A-VLPs, followed by superinfection with AA/50, were incubated. The cells collected at 48 h p.i. were subjected to immunoblotting using anti-EGFP or anti-α-actin polyclonal antibodies (C). HMV-II cells infected with WT- or C1620A-VLPs containing Luc-vRNA were lysed at the indicated periods (hrs) after infection, and the Luc activities in the respective lysates were quantified (D). The Luc activity in the WT-VLP-infected cell lysate at 12 h p.i is expressed as 100. The data obtained from three independent experiments were shown as the means ± standard deviations. Comparisons between the activities at 6, 9 and 12 h p.i. were statistically different (*p<0.05). (E) HMV-II cells infected with WT- or C1620A-VLPs were incubated at 4°C for 30 min and then transferred to 33°C, followed by incubation for a further 60 min. The cells were divided into cytoplasmic and nuclear fractions, and the GFP-vRNA contained in the respective fractions was quantified by real-time PCR. The vertical line indicates the copy number of GFP-vRNA, and the copy number in the cytoplasmic fraction of WT-VLP-infected cells at 4°C was used for normalization. The representative data from two independent experiments are shown. All comparisons between groups were statistically evaluated by using a paired *t*-test (*p<0.05; NS, not significant). Comparisons within the nuclear fractions are shown for simplicity.

A given amount of protein from the purified VLPs was subjected to immunoblotting for HEF, NP and M1 (Fig. 5A). The amount of NP contained in the C1620A-VLPs appeared to be smaller than that in WT-VLPs, a finding suggesting the presence of less GFP-vRNA in the C1620A-VLPs. We then quantified the amount of GFP-vRNA in the VLPs using real-time PCR, and found that the amount (copies per µg VLP protein) of GFP-vRNA in the C1620A-VLPs was approximately 31% of that in WT-VLPs (Fig. 5B, p<0.05). This observation indicates that the packaging of GFP-vRNA into the C1620A-VLPs occurred less efficiently than that into WT-VLPs, since there were no differences in the amount of virus proteins or GFP-vRNA expressed in the VLP-producing 293T cells (data not shown).

Next, we examined VLP-infected HMV-II cells. We analyzed HMV-II cells infected with WT- or C1620A-VLPs by flow cytometry, and found that the attachment and internalization of C1620A-VLPs occurred as efficiently as did those of WT-VLPs (data not shown). To examine reporter gene expression in the infected cells, HMV-II cells were infected with three times as much C1620A-VLP-preparation as WT-VLP-preparation, based on the difference in the amount of GFP-vRNA contained in the VLPs (Fig. 5B). At 48 h p.i., the expression level of GFP in the C1620A-VLP-infected cells was lower (30%) than that in the WT-VLP-infected cells (Fig. 5C). The difference in GFP expression in the VLP-infected HMV-II cells at 48 h p.i. was also confirmed using VLPs containing Luc-vRNA; WT-VLP:C1620A-VLP = 1.0∶0.35 (data not shown). Furthermore, the amounts of Luc expressed in the C1620A-VLP-infected cells were constantly lower than those in the WT-VLP-infected cells at 3, 6, 9 and 12 h p.i. (Fig. 5D), which is consistent with the previous observation that virus protein synthesis was reduced in the cells infected with influenza A viruses and influenza C VLPs whose uncoating was impaired [13], [15], [32]. Thus, these findings suggest that the uncoating process of C1620A-VLPs occurred less efficiently than does that of WT-VLPs.

To obtain further evidence for the impaired uncoating of the C1620A-VLPs, we quantified the incoming GFP-vRNA transported to the nucleus of VLP-infected cells according to the procedure reported previously [13], [15]. HMV-II cells were infected with VLPs so that the copy number of GFP-vRNA included in the WT-VLPs was equal to that in C1620A-VLPs; i.e., cells were infected with VLP preparations containing three times as many C1620A-VLPs as WT-VLPs. The VLP-infected cells were kept at 4°C for 30 min, transferred to 33°C, and then incubated for up to 60 min. As shown in Fig. 5(E), there was no significant difference in the total copy number of GFP-vRNA in the cells just after the incubation for 30 min at 4°C between WT- and C1620A-VLP-infected cells, indicating that an equal amount of GFP-vRNAs was used for infection. After incubation at 33°C for 60 min, the copy number of GFP-vRNA in the nuclear fraction of WT-VLP-infected cells significantly increased (p<0.05), whereas that of C1620A-VLP-infected cells was unchanged. Furthermore, after incubation for 60 min, a significant difference in the copy numbers in the nuclear fraction was observed between WT-VLP- and C1620A-VLP-infected cells (p<0.05). These findings are consistent with the hypothesis that the transport of the GFP-vRNA from the uncoated VLPs occurred less efficiently in the C1620A-VLP-infected cells than in the WT-VLP-infected cells, suggesting that the uncoating of C1620A-VLP occurs less efficiently.

## Discussion

Using an established reverse-genetics system [25], we have attempted to generate recombinant influenza C viruses lacking CM2. No infectious recombinants have been rescued to date (data not shown), suggesting that CM2 is indispensable to influenza C virus replication. We then focused on the posttranslational modifications of CM2, and generated recombinant influenza C viruses lacking CM2 palmitoylation (rC65A) and CM2 glycosylation (rN11A) [14], [15]. The rC65A virus grew as efficiently as did the rWT virus, whereas rN11A grew less efficiently than did rWT. Taking these findings together with the observations regarding influenza C VLPs lacking CM2 glycosylation, we reported that CM2 glycosylation is involved in the uncoating and packaging processes. However, the role(s) of the CM2 ion channel function (see below) in virus replication remains to be clarified.

The three cysteines at residues 1, 6 and 20 in the extracellular domain of CM2 are evolutionarily conserved [17], [18], and the cysteines are involved in multimer formation and stability of CM2 [19]. Ion channel activities associated with CM2 have been reported [11], [12], [16] (Muraki Y, Chizhmakov IV, Ogden DC, Hay A, unpublished data). A peptide corresponding to the CM2 transmembrane region forms an α-helical structure, and the CM2 transmembrane portion forms a left-handed coiled-coil tetramer [33]–[35]. Based on these findings, it is highly likely that the tetramer form of CM2 functions as an ion channel, like the influenza A virus M2 protein [36]. In the present study, therefore, we generated and analyzed a recombinant influenza C virus lacking CM2 disulfide-bond formation (rC1620A) to obtain further insights into the role(s) of CM2 in virus replication with respect to its channel function.

The rC1620A virus grew less efficiently than did the rWT virus (Fig. 1A, B), and the tetramer form of the mutant protein CM2-C1620A synthesized in the rC1620A-infected cells was present at less than trace amounts (Fig. 2A), although the dimer form of the mutant was detected even in the absence of DSP (Fig. 2B). These observations suggest that CM2 oligomerization affects virus growth; CM2 tetramerization is required for efficient virus replication or, alternatively, it is possible that CM2 dimer formation is important to or CM2 monomers inhibit virus growth.

We studied rC1620A virus growth impairment by analyzing the recombinants and VLPs. Interestingly, no significant difference was observed in the amount of CM2 in the progeny virions (Fig. 4A), although there was a significant difference in the amount of surface CM2 between rWT- and rC1620A-infected cells (Fig. 3). These findings suggest that only a small proportion of CM2 expressed on the surface of the infected-cells was incorporated into the progeny virions, which is consistent with the observation for the influenza A virus M2 protein [37].

Although the difference in the amounts of the NS gene in the one-step grown virions did not reach statistical significance (Fig. 4C), there was a statistical difference in the amount of GFP-vRNA between WT- and C1620A-VLPs (Fig. 5B), suggesting that CM2 oligomerization affects genome packaging efficiency. It is conceivable that a subtle difference in the packaging efficiency in the one-step grown virions resulted in a significant difference in multi-step virus replication. The difference in the ratios of the incorporated genomes between virions (rWT:rC1620A = 1.0∶0.8, Fig. 4C) and VLPs (WT-VLP:C1620A-VLP = 1.0∶0.3, Fig. 5B) may be attributable to differences in the experimental settings. Virus proteins and genomes are spatially and temporally expressed in a defined organized fashion in virus-infected cells. In contrast, virus components are expressed under regulation by the promoter activities of the vector in VLP-producing cells. Thus, it is possible that the difference(s) in the contribution of individual virus proteins to budding and assembly between VLP-producing cells and virus-infected cells leads to the difference in the amount of the genomes in the particles.

Hongo *et al.* hypothesized that CM2-associated channel activity plays a role in facilitating the interaction of M1 with RNP, which enhances virion assembly; the CM2 protein transported to the cell surface reduces the ionic strength just beneath the viral budding site by inducing chloride ion efflux [11]. Analysis of CM2 in the progeny virions under non-reducing conditions (Fig. 4B) suggested that CM2 molecules expressed on the rWT-infected cells mainly form disulfide-linked dimers and tetramers whereas those on the rC1620A-infected cells are present as monomers and non-covalently associated dimers. Therefore, it may be speculated that dimeric CM2-C1620A (Fig. 2B) forms tetramers *via* non-covalent linkage on the rC1620A-infected cell surface and contributes to genome packaging through its channel function. Thus, the involvement of CM2 channel function in the packaging process needs to be further studied.

Stewart and Pekosz reported that a chimeric M2 protein containing the CM2 transmembrane domain has the ability to alter the cytosolic pH and partially restore infectious virus production to M2-deficient influenza A viruses [16]. Further, the CM2 protein expressed in CV-1 cells together with a pH-sensitive hemagglutinin of influenza A virus has the ability to modulate the pH of the exocytic pathway [12]. These findings suggest that the CM2 transmembrane domain is responsible for proton permeability, and that the permeability plays a role in the uncoating process of influenza A virus. In the present study, although the tetramer form of CM2 was not apparently detected in the rC1620A virions (Fig. 4), the rC1620A virus did replicate in cultured cells (Fig. 1A, B), indicating that viruses possessing CM2 dimer as a major molecular species are able to replicate. The possibility cannot be ruled out that the tetramer form of CM2 present in the rC1620A virions at less than trace levels functions as a proton channel during the uncoating process of the virus by allowing the acidification of the virion interior. Alternatively, it may also be proposed that the dimers of CM2-C1620A exist as tetramers in the virions, but they are not stable under the conditions used in our analysis, like a recombinant influenza A virus lacking M2 oligomerization [38]. Thus, a mutant CM2 protein capable of forming tetramers but not possessing channel activities would be a candidate to clarify the relationship between the proton permeability and the uncoating.

## Supporting Information

Figure S1 **Immuofluorescence of virus-infected cells.** The rWT or rC1620A virus-infected HMV-II cells were fixed with 4% paraformaldehyde at 48 h p.i., treated with (+) or without (−) 0.2% Triton X-100, and then reacted with primary (anti-HEF MAb (J14) and anti-CM2 serum) and the respective secondary antibodies (anti-mouse IgG-FITC and anti-rabbit IgG-Alexa594). The cells were observed under a microscope and photographed.
(EPS)

Figure S2 **Growth kinetics of the recombinant viruses.** HMV-II cells were infected with the recombinant viruses at an MOI of 1 and incubated at 33°C in the absence of trypsin for 5 days. The virus yield in the culture media was titrated on LLC-MK_2_ cells. The representative data from two independent experiments are shown.
(EPS)
